# Reductive Thermal Atomic Layer Deposition Process for Gold

**DOI:** 10.1021/acsmaterialsau.2c00075

**Published:** 2023-01-11

**Authors:** Anton Vihervaara, Timo Hatanpää, Heta-Elisa Nieminen, Kenichiro Mizohata, Mykhailo Chundak, Mikko Ritala

**Affiliations:** † Department of Chemistry, University of Helsinki, P.O. Box 55, HelsinkiFI-00014, Finland; ‡ Department of Physics, University of Helsinki, P.O. Box 43, HelsinkiFI-00014, Finland

**Keywords:** atomic layer deposition, gold, thin films, reducing agent, gold halide adducts, germyl dihydropyrazine

## Abstract

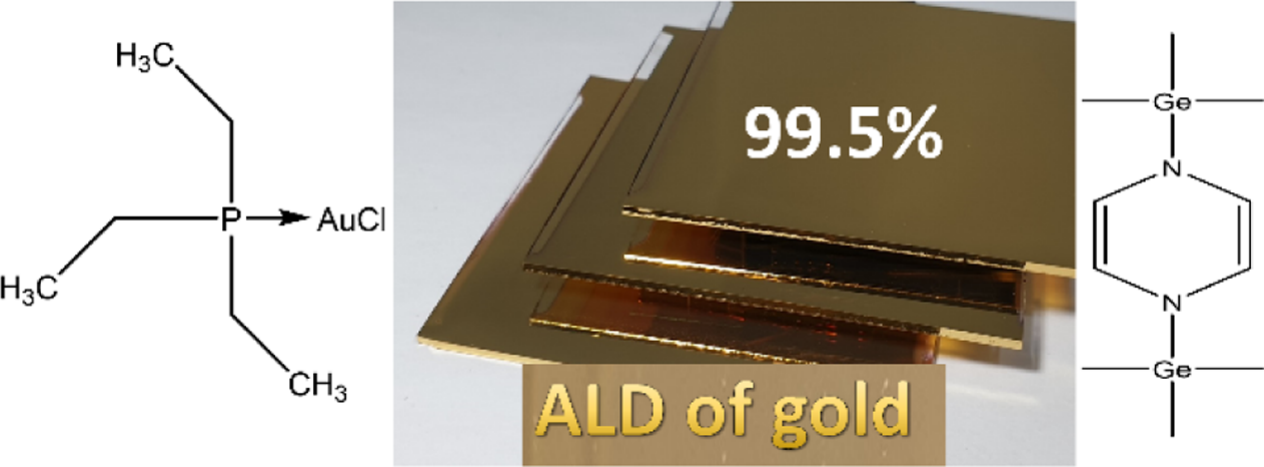

In this work, we developed an atomic layer deposition (ALD) process for gold metal thin films from chloro(triethylphosphine)gold(I) [AuCl(PEt_3_)] and 1,4-bis(trimethylgermyl)-1,4-dihydropyrazine [(Me_3_Ge)_2_DHP]. High purity gold films were deposited on different substrate materials at 180 °C for the first time with thermal reductive ALD. The growth rate is 1.7 Å/cycle after the film reaches full coverage. The films have a very low resistivity close to the bulk value, and a minimal amount of impurities could be detected. The reaction mechanism of the process is studied in situ with a quartz crystal microbalance and a quadrupole mass spectrometer.

## Introduction

Throughout the human history, gold has held a unique place among the elements, a precious metal that does not tarnish is used as a currency and in ornamentation. However, its beauty is not the only desirable trait. Gold has unprecedented chemical stability, unique catalytic properties, and the third highest conductivity of all the elements, exceeded only by silver and copper. These properties indicate high potential for gold thin films in applications such as interconnects, contact platings, biosensors, and microelectromechanical system devices.^1−4^ Furthermore, gold nanorods have been used to make localized surface plasmon resonance biosensors.^5^

For the majority of these applications, especially in microelectronics, having high-quality thin films deposited uniformly on complex three-dimensional (3D) structures is an essential requirement. Atomic layer deposition (ALD) stands as the prominent method to meet the requirements. ALD relies on saturative alternate surface reactions. Excellent thickness control and superior conformality provide uniform coatings even on complex 3D structures.^6,7^

Although several chemical vapor deposition (CVD) processes for gold exist,^8^ only three ALD processes have been reported for gold thin films. Two of the processes require hydrogen and oxygen plasma, respectively,^9,10^ and the only thermal ALD process^11^ uses ozone to combust away the ligands of the gold precursor. Thermal ALD processes for gold nanoparticles have also been reported but are not considered here in detail.^12,13^

The first gold ALD process^9^ was plasma-enhanced ALD (PEALD) and used trimethyl(trimethylphosphine)gold(III) [Me_3_Au(PMe_3_)], oxygen plasma, and water at 120 °C. The resulting gold films had some impurities, most notably carbon (6.7 at. %). The growth rate was 0.5 Å/cycle. The other PEALD process for gold^10^ used Me_3_Au(PMe_3_) with hydrogen plasma at 50–120 °C and yielded films of considerably higher purity, with the total impurity content of less than 1 at. %. The resistivity was 6 μΩ cm for the thickest films. The growth rate was 0.3 Å/cycle.

PEALD processes typically yield smooth films with little impurities, and the processes operate at low temperatures. However, possible damage to the substrates needs to be considered. Furthermore, repeatability and limited film conformality present challenges for PEALD.^14^ Thermal ALD processes do not have these problems with plasma damage and film conformality. Thermal processes are also repeatable and can be used in batch processing, and the reactor design is simpler.

The first and so far the only thermal ALD process for gold thin films used Me_2_Au(S_2_CNEt_2_) as the gold precursor.^11^ Combined with ozone, self-limiting growth was observed at a deposition temperature of 180 °C. This process had a relatively high growth rate of 0.9 Å/cycle and yielded films with resistivities of 4 μΩ cm at best. The impurity contents were also low, with 2.9 at. % of oxygen and less than 1 at. % of other elements. However, this process is highly oxidative, and thus, there is a need for reductive alternative. The best attempt to this direction so far is the combination of Au(N(SiMe_3_)_2_)(PEt_3_) and dimethylamine borane [BH_3_(NHMe_2_)] that resulted in gold deposition, but the growth rate was less than 0.10 Å/cycle, and the growth was not fully self-limiting as required in ALD.^13^

**Figure 1:**
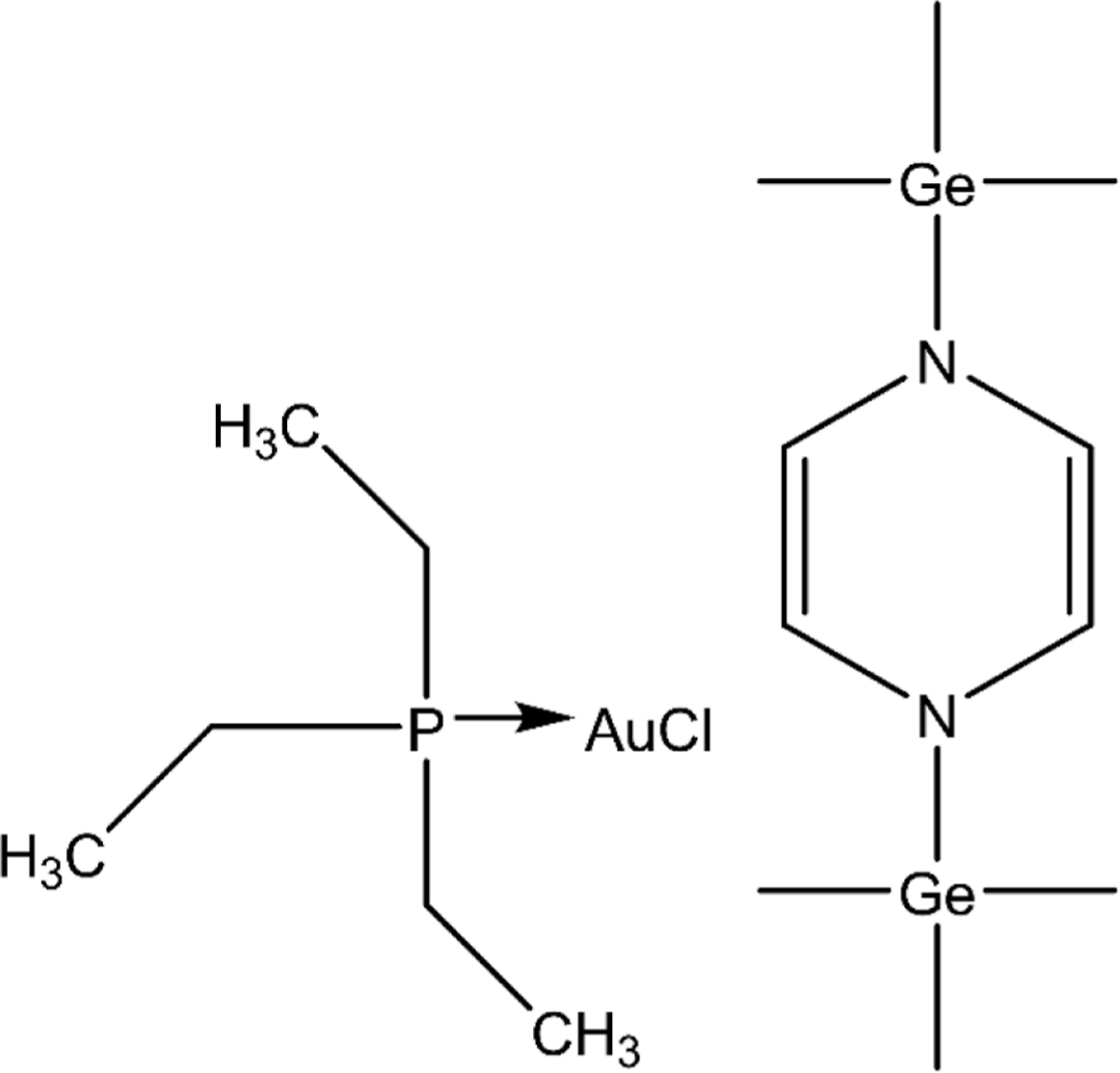
Structures of AuCl(PEt_3_) and (Me_3_Ge)_2_DHP.

In our recent research,^15^ we developed a new ALD process for nickel from novel precursors. 1,4-bis(trimethylgermyl)-1,4-dihydropyrazine ((Me_3_Ge)_2_DHP), a new reducing agent in ALD combined with dichlorobis(triethylphosphine)nickel(II) (NiCl_2_(PEt_3_)_2_), afforded high quality nickel thin films at a very low temperature of 110 °C. (Me_3_Ge)_2_DHP is analogous with the previously presented 1,4-bis(trimethylsilyl)-1,4-dihydropyrazine [(Me_3_Si)_2_DHP] reducing agent.^16,17^ The reducing capability of (Me_3_Si)_2_DHP is based on the nonaromatic dihydropyrazine being prone to oxidize and turn into aromatic pyrazine giving two electrons for reduction of, for example, metals. The reaction apparently involves a removal of the ligands L on the metal as L-SiMe_3_. Metal chlorides are especially reactive due to the thermodynamically very favorable release of alkylsilylchlorides. Our presumption is that the Me_3_Ge groups in place of the Me_3_Si groups make the compound more reactive because of the weaker Me_3_Ge–N bonds and still quite strong Me_3_Ge–Cl bonds formed in reactions with metal halides. In Ni ALD, (Me_3_Ge)_2_DHP indeed turned out to be a better reducing agent than (Me_3_Si)_2_DHP. Therefore, we proposed that (Me_3_Ge)_2_DHP could have good reactivity with other metal halides as well. In this paper, we demonstrate the first ever reductive thermal ALD process for gold thin films. In this process, a commercially available gold compound, chloro(triethylphosphine)gold(I) [AuCl(PEt_3_)], is combined with (Me_3_Ge)_2_DHP (Figure 1).

Similar to NiCl_2_(PEt_3_)_2_, also AuCl(PEt_3_) is a phosphine adduct of a metal chloride and hence expected to be reactive with (Me_3_Ge)_2_DHP. Indeed, high purity films were deposited at low temperatures of 160–180 °C. Furthermore, we investigate the reaction mechanism of the process.

## Experimental Section

### Film Deposition

Both precursor compounds, AuCl(PEt_3_) and (Me_3_Ge)_2_DHP, were synthesized in-house. Details about the reducing agent have been reported elsewhere.^15^ Details of the synthesis and thermal properties studies of AuCl(PEt_3_) are reported in the Supporting Information.

A F-120 hot-wall flow-type ALD reactor (ASM Microchemistry), with a pressure of approximately 10 mbar, was used in the experiments. The reactor uses inert gas valving to pulse the precursors. Pulse times varied from 1.0 to 6.0 s, and a purge time of 3.0 s was used for most of the depositions. N_2_ (AGA 5.0) was used as a carrier and purging gas. AuCl(PEt_3_) and (Me_3_Ge)_2_DHP were evaporated inside the reactor from open glass boats held at 160 and 45 °C, respectively. The depositions were carried out on 5 × 5 cm^2^ native oxide terminated Si substrates that were cut from wafers and on soda lime glass substrates of the same size. In addition, nucleation and film growth were studied on Ru, TiN, and Al_2_O_3_ films.

### Film Characterization

Crystal structures were studied with X-ray diffraction using a PANalytical X’Pert Pro MPD X-ray diffractometer. A grazing incidence geometry was used with the Cu Kα (λ = 1.54 Å) radiation having an incidence angle of 1°. PANalytical Highscore Plus 4.1 software was used to analyze the results.

Energy-dispersive X-ray spectrometry (EDS) was used to measure film thicknesses. An oxford INCA 350 connected to a Hitachi scanning electron microscope was used for the measurements with an electron beam voltage of 20 kV. Film thicknesses were calculated from Au M X-ray lines with the GMRFILM program using a bulk density of 19.3 g/cm^3^ for gold.^18^ These thicknesses are nominal thicknesses and do not match with island heights of noncontinuous films. However, they do indicate the amount of the deposited gold and are therefore a good measure for the overall growth. An uncertainty of 5% was estimated for the thickness measurements due to a minor fluctuation of the electron beam current and possible deviations from the bulk density.

Elemental analysis of the film composition was carried out with time-of-flight elastic recoil detection analysis (ToF-ERDA) using a 40 MeV ^127^I^7+^ ion beam. Further elemental characterization was carried out with X-ray photoelectron spectroscopy (XPS). The XPS measurements were performed with a PREVAC system having an EA-15 hemispherical electrostatic energy analyzer and a RMC50 monochromatic X-ray source with Al Kα anode (1486.7 eV). The pressure during all the measurements was 10^–10^ mbar. The analyzer pass energy was 50 eV, and the slit was C 0.3 × 25. Before the measurements, the films were rastered for 1 h with 5 keV Ar^+^ ions over an area of 5 × 2 mm to remove oxygen and other impurities from the surface of the films. The peak fitting was carried out with CasaXPS by GL line shape.

Nucleation and morphology of the films were studied with a field emission scanning electron microscope (Hitachi S-4800). Morphology and roughness of the films were studied also with atomic force microscopy (AFM, Veeco Multimode V instrument). The Si probe with a nominal tip radius of 10 nm and a spring constant of 5 N/m was used to capture images in air from films deposited on Si substrates. Images were flattened to remove artefacts from sample tilt and scanner bow. Roughness was calculated as a root-mean-square value (*R*_q_) from flattened images.

A four-point probe (CPS Probe Station, Cascade Microtech combined with a Keithley 2400 SourceMeter) was used to measure sheet resistances of films deposited on soda lime glass substrates. Sheet resistance was obtained as an average from nine measurement points uniformly distributed across the square-shaped substrate. The resistivity was calculated by multiplying the sheet resistance with the film thickness.

### Reaction Mechanism Studies

Reaction mechanism studies were carried out in a F120 SAT flow-type ALD reactor (ASM Microchemistry) to which an in situ quartz crystal microbalance (QCM) and a quadrupole mass spectrometer (QMS) are attached. Details of the specially modified ALD reactor are described elsewhere.^19^ The pressure drop from the ALD reactor (1–5 mbar) to the required QMS pressure (10^–5^ mbar) is carried out by differential pumping through a 150 μm orifice that is at the reaction temperature. The deposition area in the reactor is enlarged to produce high enough amounts of reaction byproducts for detection with QMS. The total area of the glass substrates and walls is 3500 cm^2^. The process parameters were modified to fit the special ALD reactor, and the pulse lengths of AuCl(PEt_3_) and (Me_3_Ge)_2_DHP were 10 and 12 s, respectively. The purge times after both precursors were 30 s. The sublimation temperature was 166 °C for AuCl(PEt_3_) and 47 °C for (Me_3_Ge)_2_DHP. The deposition temperature was 180 °C.

QMS measurements were carried out with a Hiden Analytical HAL/3F 510RC mass spectrometer with an ionization energy of 70 eV. QCM measurements were carried out on the gold-coated quartz crystal with Maxtek TM 400 with a sampling rate of 20 Hz. The QCM head is placed in the deposition zone between the substrates and the QMS inlet.

## Results and Discussion

### Properties of the Gold Precursor

**Figure 2:**
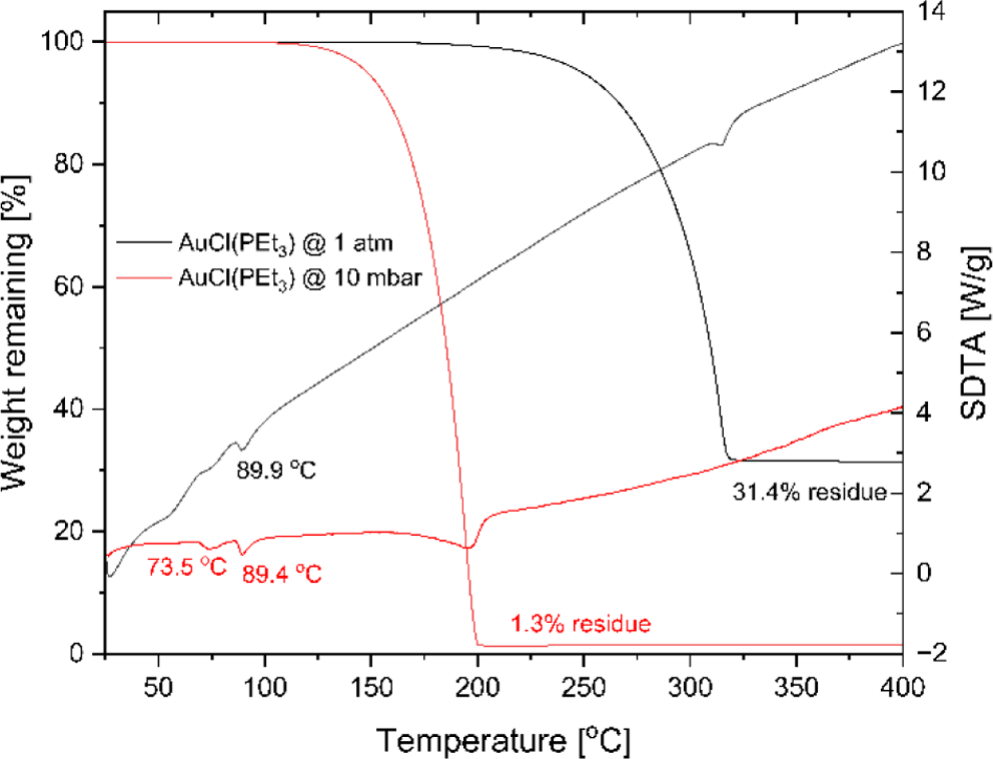
Thermogravimetric analysis and SDTA curves measured for AuCl(PEt_3_) at atmospheric pressure (black) and 10 mbar pressure (red). Measurements were carried out under a flowing N_2_ atmosphere, and heating rates of 10 and 5 °C/min were used for 1 atm and 10 mbar measurements, respectively.

Although the gold precursor used in this study, chloro(trimethylphosphine)gold(I) [AuCl(PEt_3_)], is a known compound, its suitability for ALD remained unexplored. AuCl(PEt_3_) melts at 86.5–89.9 °C and can be sublimed with a quantitative yield under vacuum at 140–160 °C/0.1 mbar. The thermogravimetric (TG) curve measured in the flowing N_2_ atmosphere at atmospheric pressure (Figure 2) shows a one-step weight loss around 200–325 °C and a residue of 31.4% while the theoretical Au content of the compound is 56.2%. This indicates that a large fraction of the compound evaporates even at atmospheric pressure. The TG curve measured in the flowing N_2_ atmosphere at 10 mbar pressure (Figure 2) shows a single-step weight loss around 100–200 °C and a residue of 1.3%, proving almost complete evaporation of the compound. Thus, it seems that in condensed form, the compound is sufficiently stable at least up to 190 °C. Vapor pressures of the AuCl(PEt_3_) compound at different temperatures were estimated using the TG method.^20^ Vapor pressures of 0.1, 0.3, 0.5, and 1 mbar are achieved at 150, 163, 169, and 178 °C, respectively. The Antoine equation derived takes the form log P(mbar) = 6.47 ± 0.04 + 0.0364 ± 0.0003 × *T*(°C).

### Gold Film Deposition and Growth Characteristics

**Figure 3:**
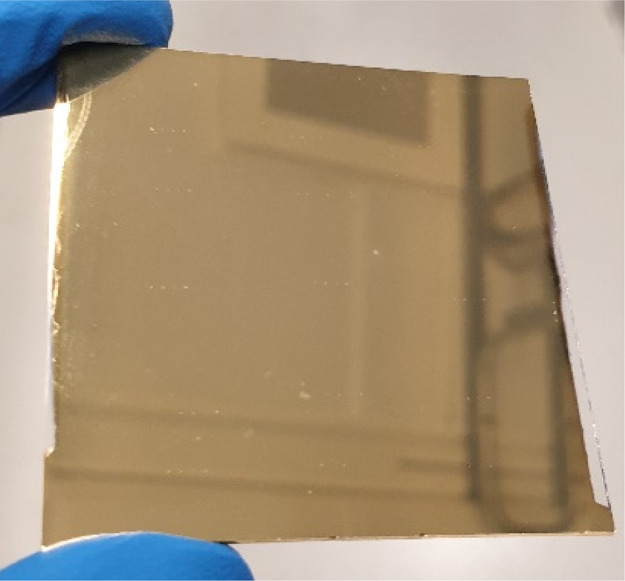
Reflective gold film deposited on soda lime glass at 180 °C.

**Figure 4:**
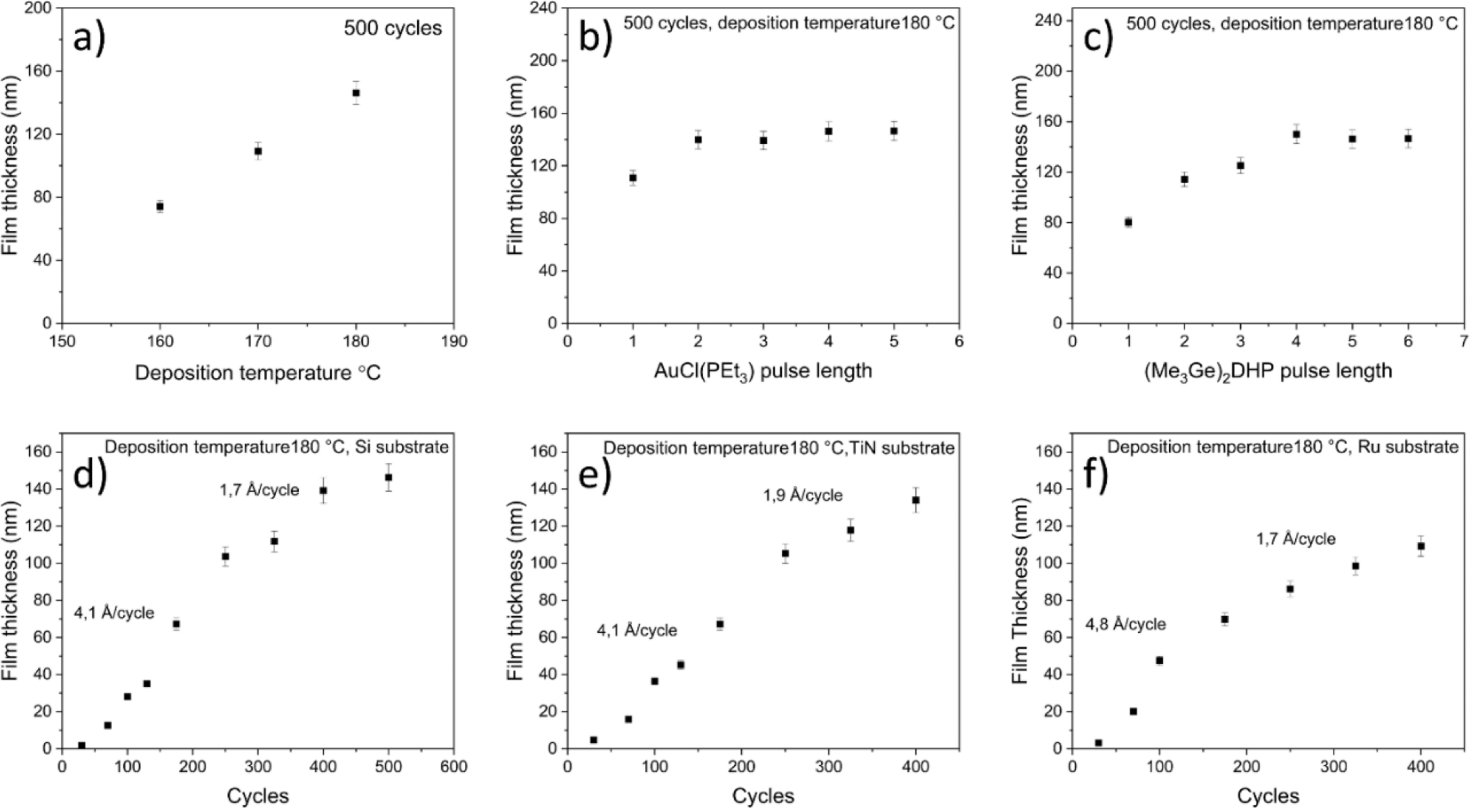
Growth characteristics of the AuCl(PEt_3_) + (Me_3_Ge)_2_DHP process. The film thickness as a function of deposition temperature (a), AuCl(PEt_3_) pulse length (b), and (Me_3_Ge)_2_DHP pulse length (c). Film thickness as a function of deposition cycles on Si (d), TiN (e), and Ru (f).

AuCl(PEt_3_) was combined with (Me_3_Ge)_2_DHP at deposition temperatures of 160–180 °C. Highly reflective gold films were obtained within this temperature range (Figure 3). The lower limit was dictated by the sublimation temperature of the gold precursor (160 °C) and the upper limit by the decomposition of the same. The decomposition of AuCl(PEt_3_) was observed visually in several experiments as formation of AuCl in the hot end of the precursor source tube where the precursor molecules have long residence time. However, despite this minor decomposition, saturation with both precursors was achieved and ALD-type growth verified. The growth characteristics of the process are presented in Figure 4. Some results are presented as film thickness instead of the more commonly used growth rate. The reason for this choice is that the process has two growth regimes with significantly different growth rates (Figure 4d–f). This is a quite typical observation in ALD of metals and will be discussed below. Calculating the growth rate by dividing the final film thickness with the total number of deposition cycles would therefore provide a value that does not correspond to either of the actual growth rates. It is emphasized that we use nominal film thicknesses that are calculated from EDS results and thereby represent the total amount of gold deposited rather than, for example, island height.

The film growth depended heavily on the deposition temperature. The film thickness almost doubled when the deposition temperature was increased from 160 to 180 °C (Figure 4a). Saturation tests were carried out at 180 °C using 500 cycles and 3.0 s purges. Self-limiting film growth was observed with 4.0 s pulses for both precursors (Figure 4b,c). The films were uniform in thickness across the substrate (Figure S1).

Film thickness as a function of number of deposition cycles was not linear, but two growth regimes were observed instead. At 180 °C, at the start of the deposition, the growth rate was approximately 4.1 Å/cycle, then decreased to 1.7 Å/cycle, and stayed constant from this point onward. As will be seen below, the change in the growth rate occurs when the films become continuous. The high growth rate at the beginning of the process can be attributed to the roughness of the films. Films that consist of islands have much larger surface area than films with smooth surfaces and thus have more adsorption sites for the precursors. When the films become continuous, the roughness decreases and so does the growth rate. However, the growth rate of 1.7 Å/cycle is still very high for a metal ALD process, being approximately 60% of a full gold monolayer thickness (2.88 Å, calculated from the lattice constants^21^). Finally, halving the purges from 3.0 to 1.5 s did not affect the growth rate.

AuCl(PEt_3_) was also tested with (Me_3_Si)_2_DHP as a reducing agent. At 180 °C, only gold islands (Figure S2) were obtained with (Me_3_Si)_2_DHP, whereas (Me_3_Ge)_2_DHP yielded a fully continuous film when the other process parameters were similar. Similarly, in our previous study^15^ on ALD of Ni from NiCl_2_(PEt_3_)_2_, only some scattered islands were achieved with (Me_3_Si)_2_DHP, whereas the combination of NiCl_2_(PEt_3_)_2_ and (Me_3_Ge)_2_DHP yielded a fully continuous nickel film.

### Film Characterization

**Figure 5:**
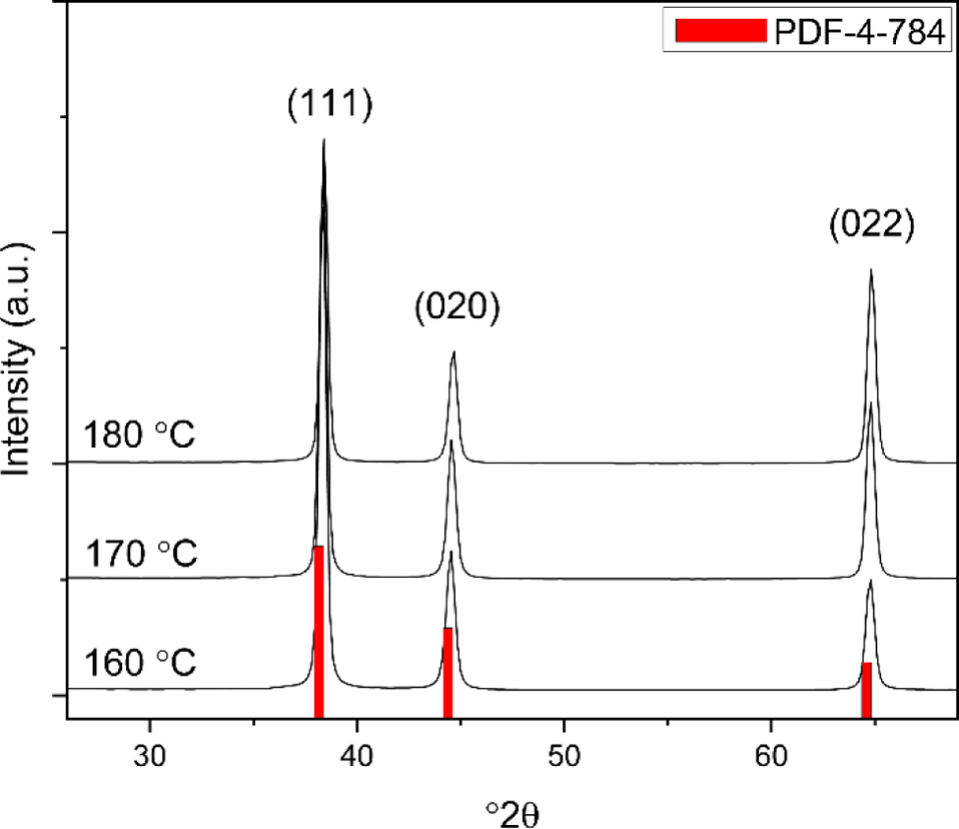
Grazing-incidence X-ray diffractogram of Au films deposited at 160–180 °C. The red bars correspond to the ICDD PDF card 4–784 for gold.

XRD measurements were carried out from films deposited at 160, 170, and 180 °C with the optimized parameters on silicon substrates. Regardless of the deposition temperature, all the diffractograms were similar and exhibited reflections matching with the cubic gold metal (Figure 5).

**Figure 6:**
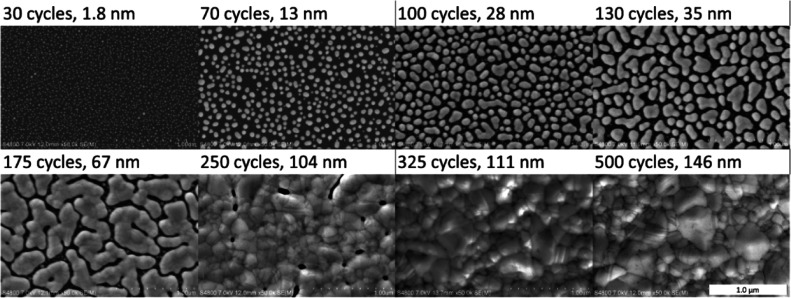
SEM images of gold films deposited with varying number of cycles at 180 °C on silicon.

**Figure 7:**
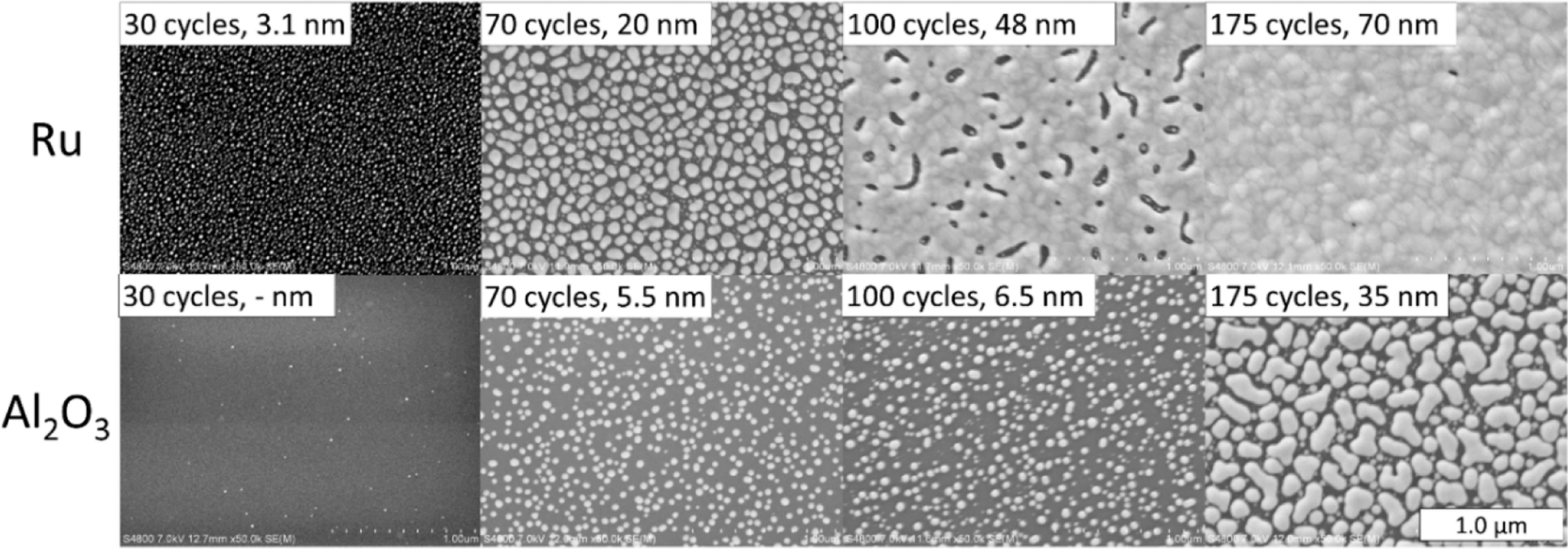
SEM images of gold films deposited with varying number of cycles at 180 °C on Ru and Al_2_O_3_ surfaces.

Nucleation and morphology of the films on the different substrates were studied with scanning electron microscopy (SEM) (Figures 6 and 7). Films were deposited on Si, TiN, Al_2_O_3_, and Ru surfaces at 180 °C with the saturative pulses. On Si, a slight nucleation delay was observed during the first 10–20 cycles (Figure 4d). However, after 30 cycles, the growth was rapid, and the process had a growth rate of approximately 4.1 Å/cycle. Up to 175 cycles, the films consisted of islands of increasing size (Figure 6). When the films became continuous after 250 cycles, the growth rate decreased to approximately 1.7 Å/cycle. While the nucleation and overall growth were similar on Si and TiN (not shown), there was a clear difference with the other two surfaces. On Ru, the nucleation density was much higher, and the films were continuous after 175 cycles corresponding to a thickness of approximately 70 nm (Figure 7). This can be attributed to better wetting characteristics of gold on another noble metal. On Al_2_O_3_, there was a clear delay in nucleation. Even though the differences between the substrates were clear, there is no difference in the type of the growth itself as the two regimes were seen on all the substrates (Figure 4d–f).

**Figure 8:**
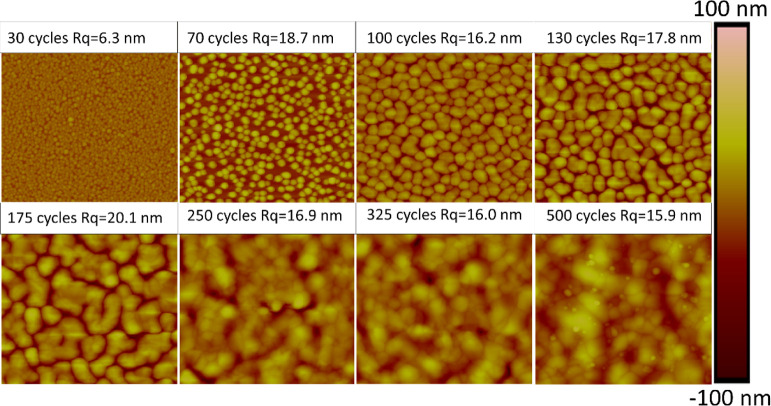
AFM images of Au films deposited with varying number of cycles at 180 °C on Si with their respective *R*_q_ values.

Morphology and roughness were also studied with AFM from the films deposited on Si at 180 °C with the saturative pulses. The root-mean-square roughness (*R*_q_) of the films was around 6.3 nm after the first 30 cycles, then sharply rose to 18–20 nm, and finally settled around 16 nm when the films became continuous (Figure 8). This dramatic increase in roughness is attributed to the strong agglomeration, as evident in the SEM and AFM images (Figures 6 and 8). Between 30 and 70 cycles, approximately 80% of the islands coalesced into larger ones, greatly increasing the surface roughness. These films are very rough when compared to other gold ALD films. Gold films deposited with hydrogen plasma had *R*_q_ of 6.5 nm.^10^ Also, the gold films deposited with the Me_2_Au(S_2_CNEt_2_)–O_3_ thermal ALD process^11^ seemed smoother in SEM images (no *R*_q_ reported) but were closer to our films than the PEALD gold films in roughness.

**Figure 9:**
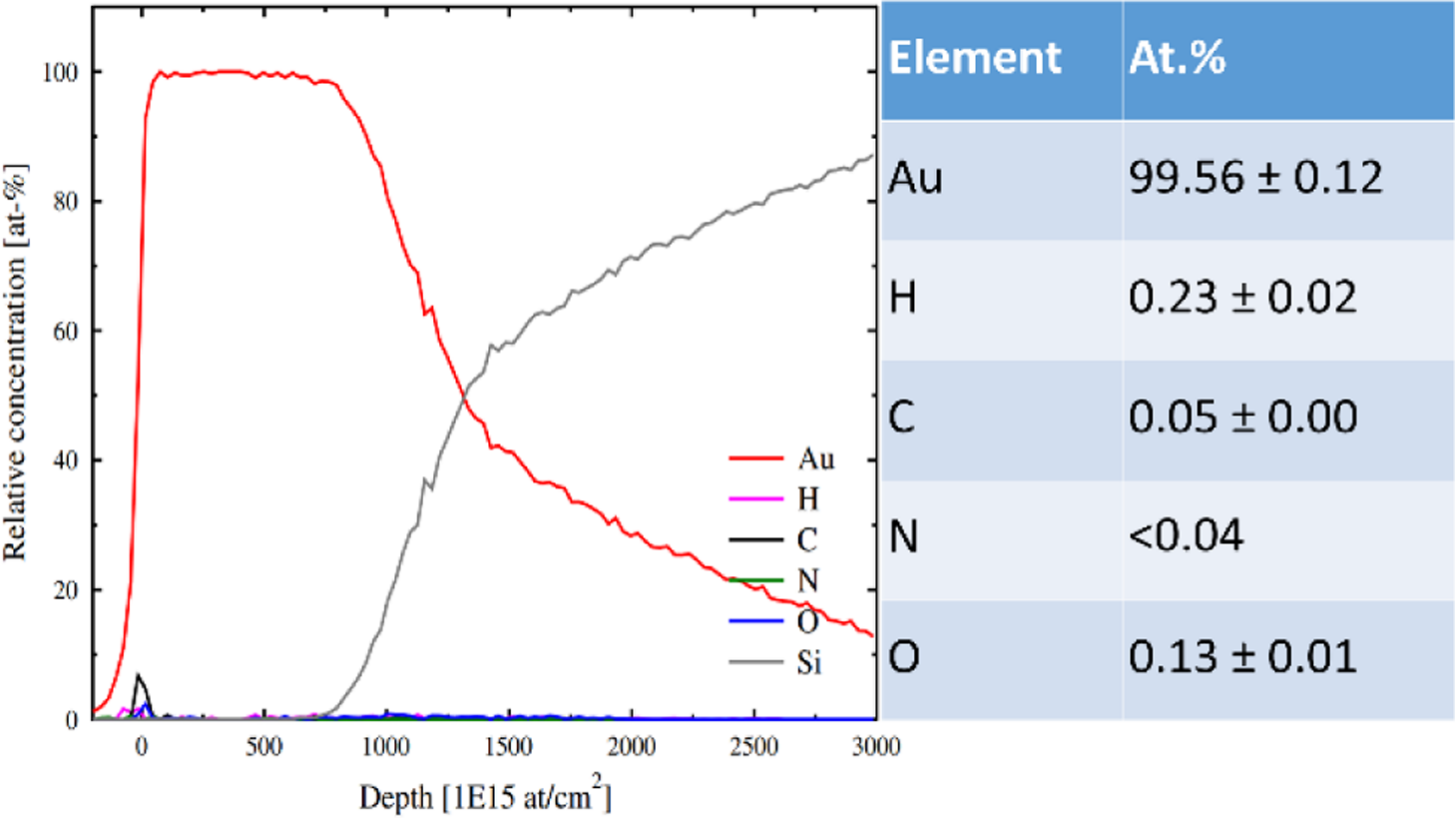
Elemental depth profiles and atomic percentages from a 145 nm Au film deposited at 180 °C.

**Figure 10:**
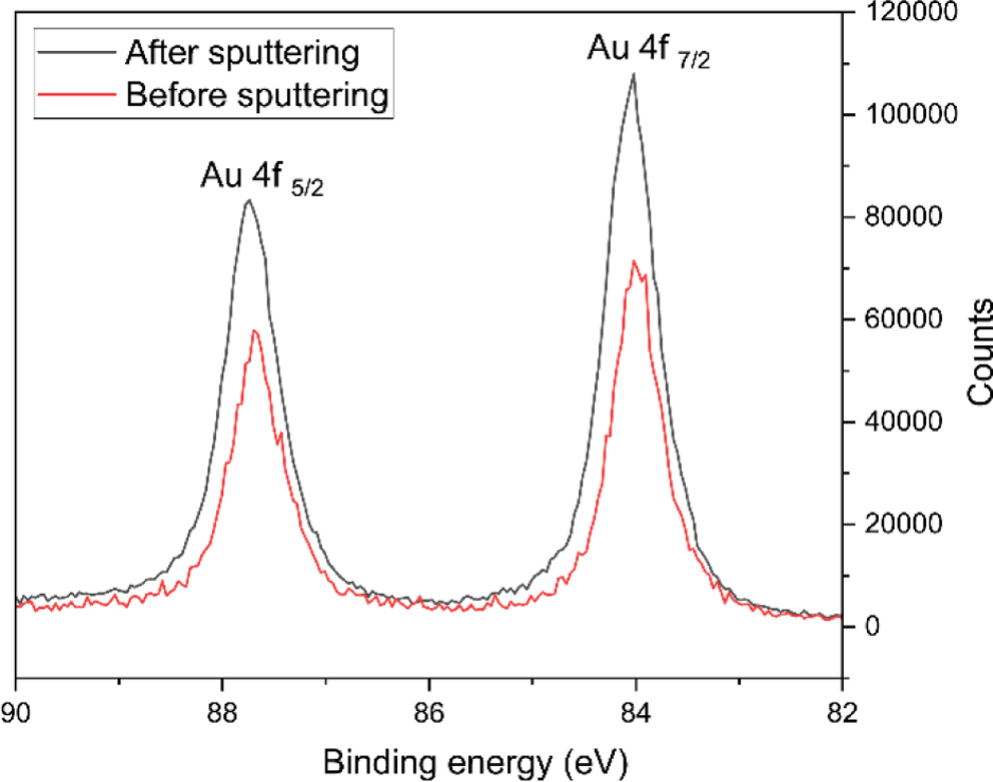
Au 4f XPS spectrum of a 145 nm thick Au film deposited at 180 °C.

Elemental composition of the films was studied with ToF-ERDA measurements (Figure 9). Films were very pure with the total amount of impurities being less than 0.5 at. %. Furthermore, majority of the impurities was found at the film surface. The high film purity indicates fast and complete reactions between the precursors. XPS measurements further confirmed the film purity as nothing else than gold could be seen after removing the contamination layer from the ambient exposure by sputtering. The Au 4f_5/2_ and Au 4f_7/2_ peaks (Figure 10) were found at 87.74 and 84.02 eV, respectively.^22^ Wide-scan spectra can be found in the Supporting Information (Figure S3).

Resistivity of the films on glass substrates was measured with the four-point probe. A fully continuous film with a thickness of 166 nm had a resistivity of approximately 2.4 μΩ cm, which is very close to the bulk resistivity of gold (2.2 μΩcm).^21^ The low resistivity was expected as the films had a large grain size and high purity.

### Mechanistic Studies

The growth mechanism was studied with a QCM and a QMS. The overall reaction is expected to proceed, as shown in reactions 1–2. A similar reaction mechanism was found for an ALD process of tin utilizing (Me_3_Si)_2_DHP and SnCl_4_.^17^ AuCl(PEt_3_) adsorbs on the surface, and the triethylphosphine adduct ligand detaches and desorbs (eq 1).
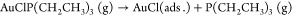
1

Next, (Me_3_Ge)_2_DHP reacts with AuCl forming gaseous byproducts (CH_3_)_3_GeCl and pyrazine and depositing Au (eq 2).
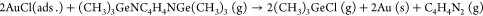
2

**Figure 11:**
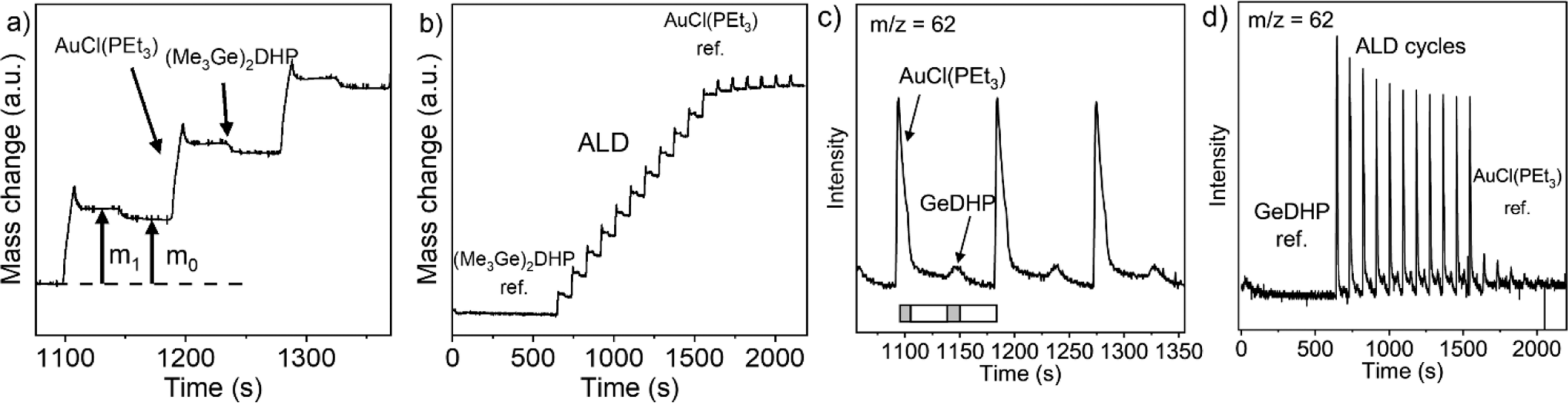
QCM measurements during (a) three ALD cycles and (b) seven (Me_3_Ge)_2_DHP reference pulses, 10 ALD cycles, and seven AuCl(PEt_3_) pulses. QMS measurement on *m*/*z* = 62 (c) during three ALD cycles and (d) during seven (Me_3_Ge)_2_DHP reference pulses, 10 ALD cycles, and seven AuCl(PEt_3_) reference pulses.

The QCM measurements show that the mass increase is rapid during the AuCl(PEt_3_) pulse, and a slight decrease occurs during the (Me_3_Ge)_2_DHP pulse (Figure 11a). The mass change m_1_ is the change after the AuCl(PEt_3_) pulse and purge. The mass change *m*_0_ is the change after a complete ALD cycle. The ratio of the mass changes *m*_1_/*m*_0_ is 1.15 ± 0.02. When the precursors were pulsed alone for reference before and after 10 ALD cycles, no mass increase was observed (Figure 11b).

With QMS, the release of the triethylphosphine adduct ligand was studied first. Figure 11c shows a close up on the QMS measurement monitoring *m*/*z* = 62, [H_2_PC_2_H_5_]^+^ originating from the P(CH_2_CH_3_)_3_ adduct ligand, during three ALD cycles. Based on all the measured *m*/*z* values (Table S1), the adduct ligand was identified to release as triethylphosphine^23^ and mostly (90%) during the AuCl(PEt_3_) pulse. When AuCl(PEt_3_) was pulsed alone, only a very slight release of PEt_3_ was detected (Figures 11d and S4). It is expected that no AuCl(PEt_3_) adsorbs on a AuCl(PEt_3_)_1–*x*_ saturated surface; hence, no PEt_3_ cleavage occurs during the reference pulses. All in all, very tiny peaks at *m*/*z* = 315 [AuPEt_3_]^+^, 118 [PEt_3_]^+^, 90 [HPEt_2_]^+^, and 62 [H_2_PEt]^+^ from AuCl(PEt_3_) were visible in QMS when the precursor was pulsed alone (Figure S4). The low intensities observed from the precursor molecule might be because of its possible condensation on the cone of the sampling orifice and because effusion fluxes of heavier molecules through the orifice to the QMS are lower than those of lighter molecules.

**Figure 12:**
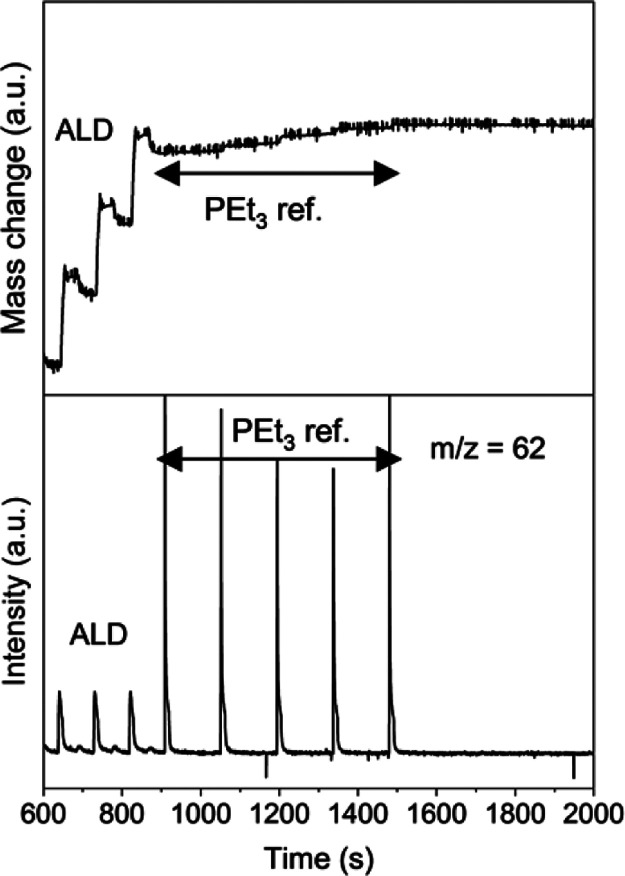
QCM (top row) and QMS (bottom row) measurements on reference pulses of triethylphosphine on the Au surface.

The behavior of the triethylphosphine adduct ligand was studied further by pulsing triethylphosphine alone on a freshly deposited Au surface (Figure 12). During the triethylphosphine pulses, a clear increase of the *m*/*z* = 62 was visible in QMS. As no mass increase was detected with QCM, it is clear that triethylphosphine does not adsorb on Au at this temperature (180 °C). AuCl(PEt_3_), on the other hand, seems to first adsorb on the surface, which is seen as a mass increase, and then desorb if the surface is already saturated with AuCl(PEt_3_)_1–*x*_ (Figure 11b). The adsorption and desorption seem to occur without a cleavage of the triethylphosphine ligand since almost no increase of the *m*/*z* = 62 is visible in QMS.

**Figure 13:**
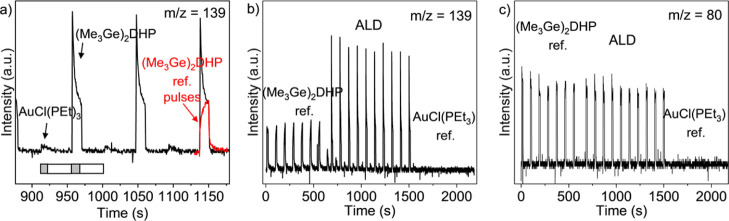
QMS measurement on (a) *m*/*z* = 139 during three ALD cycles, (b) during the whole measurement pattern of seven (Me_3_Ge)_2_DHP reference pulses, 10 ALD cycles, and seven AuCl(PEt_3_) reference pulses, and (c) *m*/*z* = 80 during the same measurement pattern. The *m*/*z* = 139 signal during (Me_3_Ge)_2_DHP reference pulses is shown as a red curve in Figure 13a.

QMS proves clearly the formation and release of the suggested reaction byproduct Me_3_GeCl. Figure 13a shows the QMS measurement on *m*/*z* = 139, [ (CH_3_)_2_^74^Ge^35^Cl]^+^, a fragment ion which has the highest intensity in the Me_3_GeCl mass spectrum,^24^ during three ALD cycles. Almost all (around 90%) of the compound is formed during the (Me_3_Ge)_2_DHP pulse. Figure 13b shows a QMS measurement on *m*/*z* = 139 during the full measurement pattern. A clear signal was visible also during the (Me_3_Ge)_2_DHP reference pulses. Presumably, the precursor or its fragments react with residual chlorine in the measurement system and create a background signal of *m*/*z* = 139 (marked also as a red curve in Figure 13a). Hence, the real intensity associated with the reaction with AuCl on the surface during the ALD cycles is the difference between the *m*/*z* = 139 intensities during the ALD process and the (Me_3_Ge)_2_DHP reference pulses. The highest peak of the mass spectrum of pyrazine^25^ (*m*/*z* = 80) was observed too (Figure 13c). Pyrazine is the byproduct of the redox reaction that produces metallic gold (eq 2), and it was seen only during the (Me_3_Ge)_2_DHP precursor pulses during the ALD cycles. A comparable intensity is observed during the (Me_3_Ge)_2_DHP reference pulses, however.

Based on the QMS measurements, during the AuCl(PEt_3_) most of the PEt_3_ desorbs, and a small amount of (CH_3_)_3_GeCl is formed in the reaction between adsorbed Me_3_Ge and chloride ligands (eqs 3 and 4).
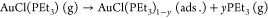
3
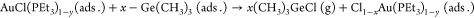
4

During the (Me_3_Ge)_2_DHP pulse, most of the chlorides react, and the rest of the PEt_3_ desorbs. Gold in AuCl is reduced by (Me_3_Ge)_2_DHP, and pyrazine and Me_3_GeCl are released as byproducts (eq 5). A small fraction of Me_3_Ge groups adsorbs onto the Au surface.
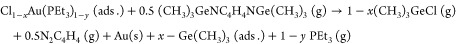
5

In reactions 3–5, *y* and *x* are approximately 0.9 and 0.1, respectively. The overall reaction is the sum of the reactions that take place during each pulse (eq 6).
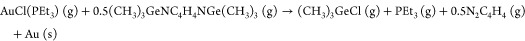
6

The QMS and QCM results can be compared by calculating the *m*_1_/*m*_0_ ratio based on the QMS results and comparing that with the *m*_1_/*m*_0_ measured with QCM. After the AuCl(PEt_3_) pulse, the overall mass change on the surface is *m*_1_ = *M*(AuCl(PEt_3_)) – *x* × *M*(Me_3_GeCl) – *y* × *M*(PEt_3_). After the (Me_3_Ge)_2_DHP pulse, that is, full ALD cycle, metallic Au is deposited and *m*_0_ = *M*(Au). The ratio *m*_1_/*m*_0_ calculated using the molar masses of the compounds and elemental gold with the *x* and *y* obtained from QMS is approximately 1.16 (eq 7). The result agrees well with the *m*_1_/*m*_0_ of 1.15 ± 0.02 measured with the QCM.
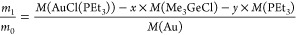
7

However, these calculations are only to support the qualitative observations of the reaction mechanism. In fact, the *m*_1_/*m*_0_ ratio is heavily dominated by the gold containing species because the *M*(AuCl)/*M*(Au) = 1.18.

## Conclusions

We successfully developed the first reductive thermal ALD process for elemental gold using AuCl(PEt_3_) and (Me_3_Ge)_2_DHP as precursors. Highly conductive and pure gold films could be deposited at moderate temperatures of 160–180 °C. The process was proven to work on multiple substrates, although with a clear difference in nucleation that was the most favorable on a Ru surface and the least favorable on Al_2_O_3_. Furthermore, the reaction mechanism was studied and found to proceed stepwise, as expected based on the literature. The combination of high growth rate and purity of the films shows potential for many applications and furthermore proves the capabilities of the recently discovered reducing agent, (Me_3_Ge)_2_DHP.

## Supporting Information Available

Synthesis of the gold precursor, film uniformity as a function of distance from the inlet area, SEM from the gold film deposited with AuCl(PEt_3_) and (Me_3_Si)_2_DHP, wide-scan XPS spectra of a gold film, all measured *m*/*z* values, and QMS measurements of AuCl(PEt_3_) (PDF)
