# Supplementary material for: Reductive Thermal Atomic Layer Deposition Process for Gold
Source: ACS Mater Au. 2023 Jan 11;3(3):206–14. doi: 10.1021/acsmaterialsau.2c00075 (PMC10176613; doi:10.1021/acsmaterialsau.2c00075)
Supplement: Supplementary file 1 — mg2c00075_si_001.pdf [file mg2c00075_si_001.pdf]

Reductive thermal atomic layer deposition process for gold

Anton Vihervaara[*,†], Timo Hatanpää[†], Heta-Elisa Nieminen[†], Kenichiro Mizohata[‡], Mykhailo Chundak[†], Mikko Ritala[*,†]

[†] Department of Chemistry, University of Helsinki, P.O. Box 55, FI-00014 Helsinki, Finland
[‡] Department of Physics, University of Helsinki, P.O. Box 43, FI-00014 Helsinki, Finland

Supporting information

Table of Contents

Synthesis of the gold precursor

Figure S1: Film uniformity as a function of distance from the inlet area

Figure S2: SEM from Gold film deposited with $AuCl(PEt_3)$ and $(Me_3Si)_2DHP$

Figure S3. Wide-scan XPS spectra of a gold film

Table S1: All measured m/z values

Figure S4: QMS measurements of $AuCl(PEt_3)$

Synthesis of the gold precursor

Synthesis and all manipulation of the compound were done under rigorous exclusion of air and moisture using Schlenk techniques and argon filled glove box. Gold (kultakeskus, 99.9%), tetrahydrohtiophene (Aldrich, 99%), 10% triethylphosphine in hexane (ABCR, 99%) and ethanol (VWR Chemicals, 96%) were used as received. THF was distilled freshly from sodium benzophenone ketyl radical and sodium metal. TGA was done using a Mettler Toledo TGA/DSC 3+ equipment. Measurements were done under flowing N_2 atmosphere at 1 atm and 10 mbar pressures. The 9 – 11 mg samples were heated from 25 – 400 °C with heating rates of 10 °C/min (1 atm) and 5 °C/min (10 mbar). NMR spectra were measured using a Bruker Avance 400 MHz spectrometer. ^1H and ^{13}C shifts are referenced to internal solvent resonances and reported in parts per million relative to TMS. Mass spectrum was recorded with a Jeol JMS-700 spectrometer operating in electron impact mode (70 eV) using a direct insertion probe.

Synthesis of Chloro(triethylphosphine)gold(I). 10 g (50.77 mmol) of gold was dissolved in aqua regia (1:3, HNO_3(70%) /HCl (37%)). The solution was heated into dryness and dried in vacuum at 70 °C for 2 hours. 250 ml oh EtOH (96%) and 25 ml of H_2O were added to the resulting red-orange $HAuCl_4$. To this solution through a dropping funnel 17.91 g (169.10 mmol) of tetrahydrothiophene was added dropwise causing the precipitation of a white solid. Stirring was continued for 1 h and then the reaction mixture filtered. The precipitate was washed sequentially with 2 x 30 mL of ethanol, 2 x 30 mL of diethyl ether, and then dried

under high vacuum for 16 hours. Yield for the chloro(tetrahydrothiophene)gold(I) was 14.905 g (91.5%). ^{1}H NMR ($CDCl_3$, 400.14 MHz, 25°C): δ = 2.24 (s, 4H, CH_2), 3.48 (s, 4 H, CH_2) ppm. ^{13}C NMR ($CDCl_3$, 100.61 MHz, 25°C): δ = 30.66 (s,CH_1), 40.52 (s, CH_2S) ppm. Chloro(tetrahydrothiophen)gold(I) (14.75g, 46.01 mmol) was then put into 350 ml Schlenk-bottle and 200 ml THF was added. To this suspension 54.40 g 10 wt% triethylphosphine in hexane, equal with 5.44g (46.01 mmol) of pure PEt_3, was added dropwise. During the addition white solid dissolved. The reaction was stirred for 1 hour. Solution was filtered with a Schlenk sinter through a Celite pad. The precipitate was washed with 2 x 30 ml THF. Filtrate was evaporated to dryness to give a white solid. Yield 16.13 g (100 %). ^{1}H NMR ($CDCl_3$, 400.14 MHz, 25°C): δ = 1.17 (t, 4H, CH_3), 1.22 (t, 5H, CH_3), 1.84 (m, 6H, CH_2) ppm. ^{13}C NMR ($CDCl_3$, 100.61 MHz, 25°C): δ = 9.12 (s, CH_3), 18.04 (s, CH_2), 18.41 (s, CH_2) ppm. *m/z* (EI, 70eV) 350 $[AuClPEt_3]^+$, 315 $[AuPEt_3]^+$, 286 $[AuPEt_2]^+$, 270 [AuPEtCH], 258 $[AuPEtH]^+$, 243 $[AuPCH_3]^+$, 118 $[PEt_3]^+$, 103 $[PEt_2CH_2]^+$, 90 $[PEt_2H]^+$, 62 $[PEtH_2]^+$.

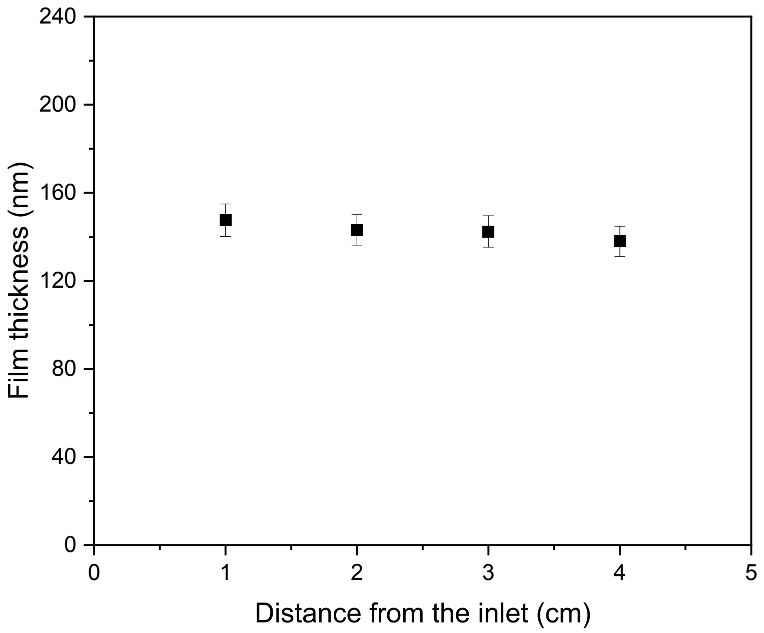

Figure S1. Thickness of the gold film across the substrate. 500 deposition cycles at 180 °C. 4.0 s pulses for $AuCl(PEt_3)$ and $(Me_3Ge)_2DHP$, 3.0 s purges.

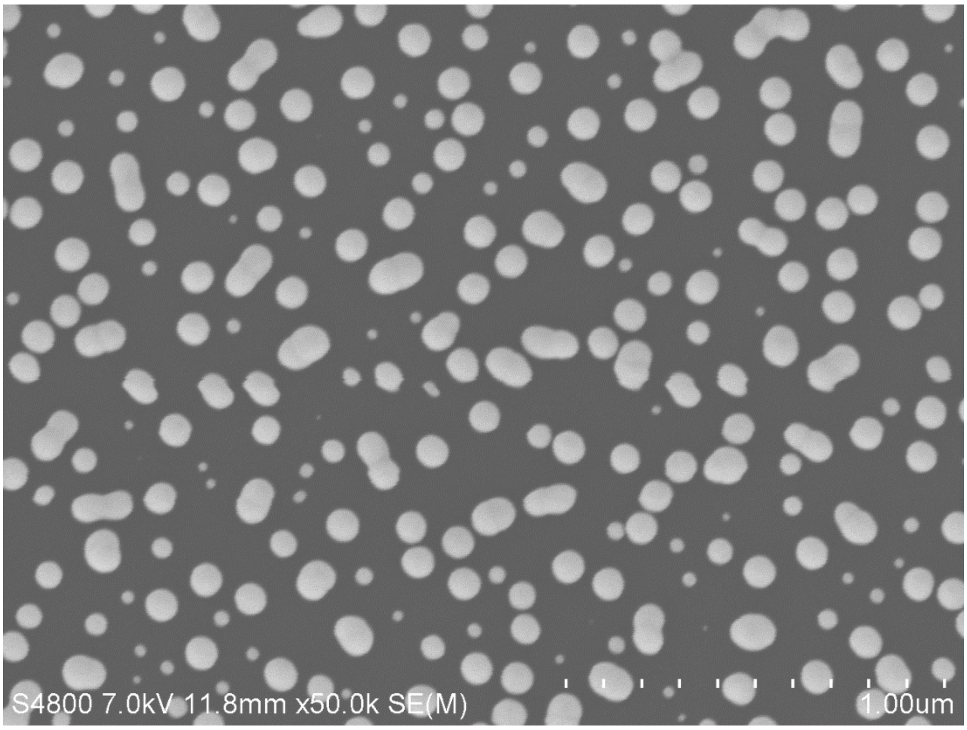

Figure S2. Gold islands deposited with AuCl(PEt_3) and $(Me_3Si)_2$DHP at 180 °C. 500 cycles, with 4 s pulse of Au(PEt_3), 3 s pulse of $(Me_3Si)_2$DHP, and purges of 3 s.

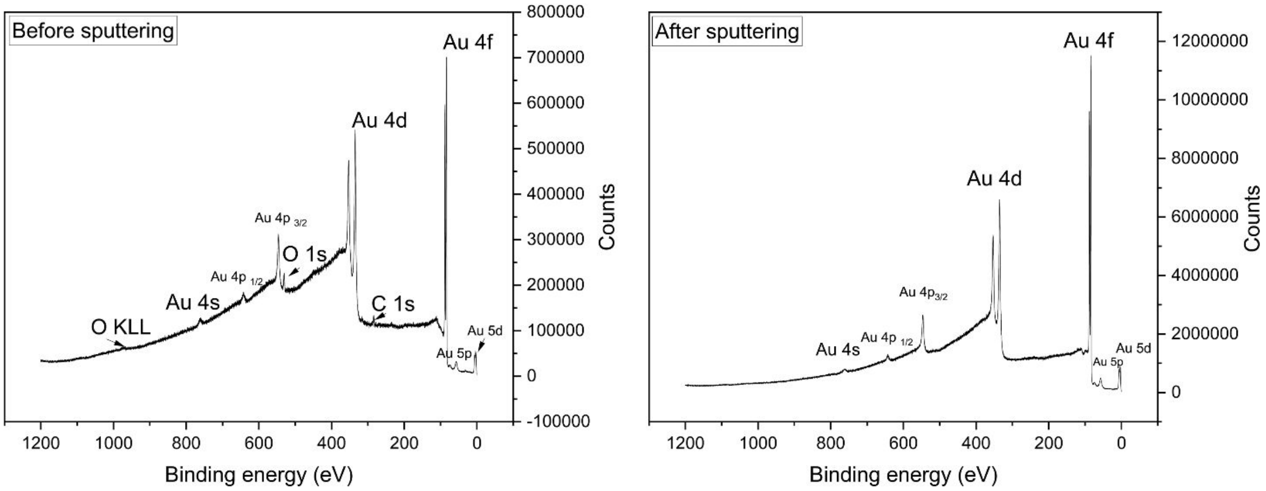

Figure S3. Wide-scan XPS spectra of a gold film deposited at 180 °C before (left) and after (right) surface cleaning by sputtering.

Table S1. The m/z values measured, observations if they were detected, the possible fragment and the suggested compound they originate from.

m/z	observation	Possible fragment	Suggested compound
118	yes	$[P(C_2H_5)_3]^+$	$P(CH_2CH_3)_3$ (ref.1)
103	yes	$[(H_5C_2)_2PCH_2]^+$	
90	yes	$[HP(C_2H_5)_2]^+$	

62	yes	$[H_2PC_2H_5]^+$	
61	yes	$[HPC_2H_5]^+$	
59	yes	$[PC_2H_4]^+$	
154	low amount	$[^{74}Ge^{35}Cl(CH_3)_3]^+$	$(CH_3)_3GeCl$
139	yes	$[^{74}Ge^{35}Cl(CH_3)_2]^+$	
137	yes	$[^{72}Ge^{35}Cl(CH_3)_2]^+$	
135	yes	$[^{70}Ge^{35}Cl(CH_3)_2]^+$	
124	low amount	$[^{74}Ge^{35}ClCH_3]^+$	
122	low amount	$[^{72}Ge^{35}ClCH_3]^+$	
119	yes	$[^{74}Ge(CH_3)_3]^+$	
117	yes	$[^{74}Ge(CH_3)_3]^+$	
109	yes	$[^{74}Ge^{35}Cl]^+$	
89	yes	$[^{74}GeCH_3]^+$	
87	yes	$[^{72}GeCH_3]^+$	
80	yes	$[C_4H_4N_2]^+$	$C_4H_4N_2$ (ref. 2)
53	yes	$[C_3H_3N]^+$	

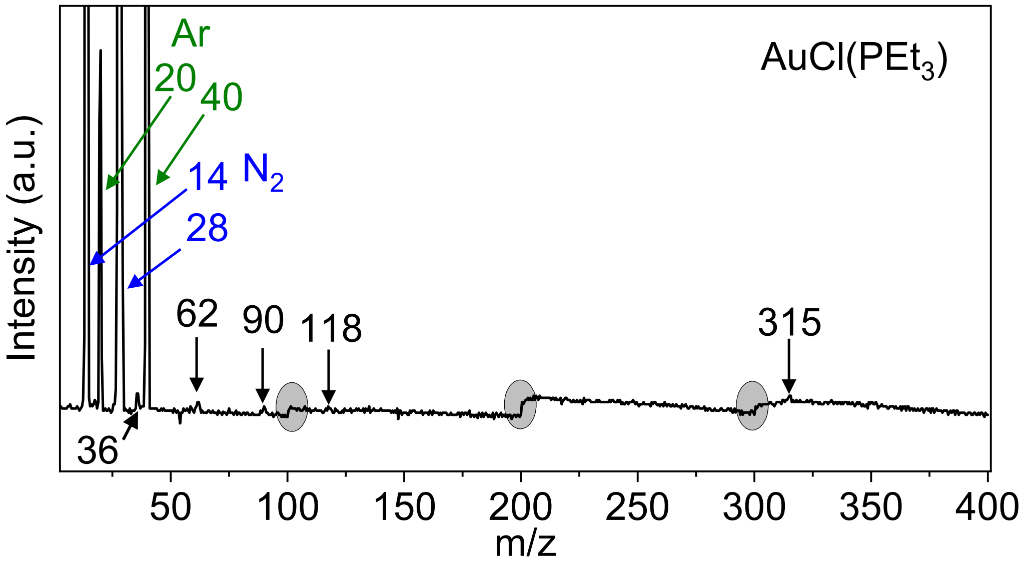

Figure S4. $AuCl(PEt_3)$ was pulsed alone for reference at the same time scanning from m/z = 0 to 400. Regions from m/z = 0-100, 100-200, 200-300 and 300-400 were recorded individually with a fresh pulse to ensure quality data and high enough dose of $AuCl(PEt_3)$ for each measurement. The background level increases whenever new measurement starts (marked with a gray circle). Signals from $AuCl(PEt_3)$ (m/z 62, 90, 118 and 315) were observed during the pulsing. Ar (m/z 40 and 20), N_2 (m/z 28 and 14) and m/z 36 (presumably from HCl) were seen constantly in the chamber/in QMS as residual gases.

References

[1] Wada, Y.; Kiser, R. W. A Mass Spectrometric Study of Some Alkyl-Substituted Phosphines1. *J. Phys. Chem.* **1964**, *68* (8), 2290–2295. https://doi.org/10.1021/j100790a044.

[2] Wallace, W. E. 'Mass Spectra' by NIST Mass Spectrometry Data Center. in *NIST Chemistry WebBook, NIST Standard Reference Database Number 69* (eds. Linstrom, P. J. & Mallard, W. G.) (National Institute of Standards and Technology). doi:https://doi.org/10.18434/T4D303
